# Suicidal thoughts, suicide attempt and non-suicidal self-harm amongst lesbian, gay and bisexual adults compared with heterosexual adults: analysis of data from two nationally representative English household surveys

**DOI:** 10.1007/s00127-023-02490-4

**Published:** 2023-06-09

**Authors:** Garrett Kidd, Louise Marston, Irwin Nazareth, David Osborn, Alexandra Pitman

**Affiliations:** 1 UCL Division of Psychiatry, Maple House, 149 Tottenham Court Road, London, W1T 7NF UK; 2 UCL Research Department of Primary Care and Population Health, Upper Third Floor, UCL Medical School (Royal Free Campus), Rowland Hill Street, London, NW3 2PF UK; 3 Camden and Islington NHS Foundation Trust, St Pancras Hospital, St Pancras Way, London, NW1 0PE UK

**Keywords:** Suicide, Self-harm, Sexual minority, Gay, Lesbian, Bisexual, Heterosexual, LGB

## Abstract

**Purpose:**

We aimed to compare differences in suicidality and self-harm between specific lesbian, gay and bisexual (LGB) groups, and investigate whether minority stress factors might contribute to any associations, addressing methodological limitations of previous research.

**Methods:**

We analysed data combined from two population-based representative household surveys of English adults (*N* = 10,443) sampled in 2007 and 2014. Using multivariable logistic regression models adjusted for age, gender, educational attainment, area-level deprivation, and common mental disorder, we tested the association between sexuality and three suicide-related outcomes: past-year suicidal thoughts, past-year suicide attempt, and lifetime non-suicidal self-harm (NSSH). We added bullying and discrimination (separately) to final models to explore whether these variables might mediate the associations. We tested for interactions with gender and survey year.

**Results:**

Lesbian/gay people were more likely to report past-year suicidal thoughts [adjusted odds ratio (AOR) = 2.20; 95% CI 1.08–4.50] than heterosexuals. No minority group had an increased probability of suicide attempt. Bisexual (AOR = 3.02; 95% CI = 1.78–5.11) and lesbian/gay (AOR = 3.19; 95% CI = 1.73–5.88) individuals were more likely to report lifetime NSSH than heterosexuals. There was some evidence to support a contribution of bullying in the association between lesbian/gay identity and past-year suicidal thoughts, and of each minority stress variable in the associations with NSSH. There was no interaction with gender or survey year.

**Conclusion:**

Specific LGB groups are at elevated risk of suicidal thoughts and NSSH, with a possible contribution of lifetime bullying and homophobic discrimination. These disparities show no temporal shift despite apparent increasing societal tolerance towards sexual minorities.

**Supplementary Information:**

The online version contains supplementary material available at 10.1007/s00127-023-02490-4.

## Introduction

Suicide is one of the leading causes of premature death worldwide, accounting for over 700,000 reported deaths annually [1, 2]. High-quality registry-based studies indicate that minority sexual orientation is a risk factor for suicide [3]. Approximately 5500 people die by suicide annually in the UK [4], but routine mortality statistics are not broken down by sexuality (or ethnicity). This hampers our understanding of suicide risk in relation to minority status in Britain, despite sexual minority groups constituting 3.2% of the England and Wales populations [5]. A body of evidence supports an excess risk of mental health problems in sexual minority groups compared with heterosexuals [6], but there is less contemporary research comparing their self-harm and suicide-related outcomes. This is despite an estimate, based on UK survey data, that 65% of gay men and 48% of lesbians who reported ever having harmed themselves cited their sexual orientation as wholly or partly the motive [7].

The UK is a heteronormative society where lesbian, gay and bisexual (LGB) people have experienced systemic and historical persecution under British law [8]. Minority stress theory suggests that experiences of discrimination [9] or of being bullied [10] may account for mental health disparities between sexual minorities and heterosexual peers [11]. More recent cultural and legal changes in the UK have provided LGB people with greater legal protection under the Equality Act 2010, and surveys of British attitudes over this period suggest greater tolerance of same-sex relationships [12]. It is unclear, however, whether this has translated into reductions in self-harm or suicidality, where self-harm disparities between heterosexuals and sexual minority groups are apparent from early on in adolescence [13].

Systematic reviews identify clear gaps in the literature regarding self-harm and suicidality risk in sexual minority groups in the UK. A 2008 systematic review of research published up to 2005 described a twofold elevated risk of lifetime suicidal ideation and of lifetime suicide attempts in lesbian, gay and bisexual (LGB) people, although the quality of included studies was limited [14]. Only one eligible study was UK based, finding an excess risk of lifetime self-harm in gay men and lesbians compared with heterosexuals, but this used non-random sampling and did not delineate the self-harm measure used [7]. A 2017 systematic review and meta-analysis of studies on adolescents and young adults found an excess risk of suicide attempts in sexual minority adolescents and youths (over twice the risk), particularly amongst gay or bisexual men [15]. Of the 14 included studies, only 1 was UK based, and this schools-based survey found no association between sexuality and risk of suicide attempt but an excess risk of non-suicidal self-injury [16]. Methodological problems identified by the systematic review authors included issues over selection of confounders [15]. A 2022 systematic review and meta-analysis of population-based studies found an almost tripling of the risk of suicidality (aggregating suicidal ideation and/or suicide attempt) in lesbian women and gay men when compared to heterosexuals, and almost five times the risk for bisexual individuals [17]. These findings derived from only eight eligible studies investigating suicidality (none in the UK), with authors noting that studies of a higher methodological quality were less likely to find disparities in risk of suicidality [17]. Methodological limitations of the eight studies related to statistical power, sexuality definitions and selection of confounding variables. Findings also did not separate out suicidal thoughts from self-harm.

This appraisal of published evidence indicates a clear need for high-quality contemporary, UK population-based research that distinguishes between individual sexual minority groups, separates out specific suicidality outcomes, overcomes sampling methodology limitations and adjusts for appropriate confounders. It provides a rationale for the current investigation, the overarching aim of which was to understand the burden of suicidality and self-harm amongst sexual minority groups in the UK, identify likely risk factors, and ascertain whether any inequalities in suicidality have narrowed or widened over the period 2007–2014. In this study, we aimed to address the limitations of previous research by analysing data from a representative household sample with adequate statistical power to compare specific sexual orientation minority groups in the UK. Our objectives were:

- To describe the proportion of UK household members self-identifying as each sexual orientation group in 2007 and 2014, and their socio-demographic and clinical characteristics
- To describe associations between specific LGB sexual orientation and suicidality (past-year suicidal thoughts, past-year suicide attempt and lifetime non-suicidal self-harm), controlling for common mental disorder.
- To examine the influence of minority stress indicators (bullying and discrimination) on associations between specific LGB sexual orientation and suicidality
- To test whether associations between specific LGB sexual orientation and suicidality differed by gender and by survey year.

## Methods

### Sample

We conducted a secondary analysis of data from two separate survey years of the Adult Psychiatric Morbidity Survey (APMS) for England: 2007 [18] and 2014 [19]. APMS was the first national government health survey in the UK to include questions on sexual orientation, also collecting data on common mental disorder (CMD), past trauma, and a rich set of socio-economic (education, housing and employment) and health-related variables [20].

The APMS uses a stratified, multistage random sampling design, selecting residential addresses within selected primary sampling units and one adult from each address, to create a sample representative of the population aged 16 and over living in private households in England [20, 21]. Eligible participants need to speak English to a level sufficient for being interviewed in English. Data are collected using computer-assisted face-to-face interviewing, including structured diagnostic assessments and screening instruments for mental disorders, supplemented by computer-assisted self-interviewing (CASI) for sensitive questions (such as sexuality, suicidality, drug and alcohol use, abuse and discrimination) to promote disclosure.

We combined data from two successive APMS surveys to create a probability sample of the English population aged 16 and over. As the sexuality question was excluded for participants aged 65 and over in the 2014 survey, due to perceived question burden [22], we included in our analysis individuals aged 16–64 who had specified sexuality in the 2007 and 2014 surveys (*n* = 10,443).

### Ethical approval

The Royal Free Hospital and Medical School Research Ethics Committee (reference number 06/Q0501/71) provided ethical approval for AMPS 2007. The West London National Research Ethics Committee (reference number 14/LO/0411) provided ethical approval for AMPS 2014.

## Measures

### Exposure

Our exposure of interest was sexual orientation. As this was defined slightly differently in the surveys for 2007 and 2014, we harmonised those definitions to create four categories, as per our previous analysis of these datasets [23], providing the most valid comparison across the two datasets: heterosexual and mainly heterosexual (reference group); bisexual; lesbian/gay and mainly homosexual; and other.

### Outcomes

We investigated three binary outcomes capturing suicidality and self-harm [24, 25]:

1. Past-year suicidal thoughts: based on responses to a face-to-face question “Have you ever thought of taking your life, even though you would not actually do it?” and specifying when this last occurred.
2. Past-year suicide attempt: based on a face-to-face and CASI question “Have you ever made an attempt to take your life, by taking an overdose of tablets or in some other way? and specifying when this last occurred.
3. Lifetime non-suicidal self-harm (NSSH): based on a face-to-face and CASI question ‘Have you ever deliberately harmed yourself but not with the intention of killing yourself?’. Use of a lifetime measure captured longer term self-harming patterns over the developmental course of sexual identity milestones [26].

### Covariates

We chose the following covariates as potential confounders based on clinical experience and published evidence:

- Self-reported age
- Gender (self-identified; male/female)
- Highest educational attainment
- Area-level deprivation using the Index of Multiple Deprivation (IMD); a composite index of relative deprivation at small area level, based on seven indicators of deprivation: income; employment; health deprivation and disability; education, skills and training; barriers to housing and services; crime and disorder; and living environment [27]. Each respondent’s postcode was used to link to the corresponding deprivation quintile (1 denoting least deprived).
- Common mental disorder (CMD) based on the Revised Clinical Interview Schedule (CIS-R): Questions on CMD were administered using a structured interview schedule examining the existence of non-psychotic CMD symptoms (depression and anxiety) in the week prior to interview [28]. A score of 12 or more indicates that the individual meets the CIS-R threshold for a level of CMD symptoms that warrants primary care intervention. As previously, we, therefore, used this to denote the presence of CMD [23].
- Survey year (2007/2014), to capture any potential changes over time and to consider sexual orientation data collection differences.

### Putative mediators

To test for evidence of possible mediation, we selected the following minority stress variables to add to final models, on the assumption that any variable acting as a mediator would be expected to reduce the magnitude of any association observed:

- Past-year discrimination based on sexual orientation using a binary measure based on CASI responses to the question: "Have you been unfairly treated in the last 12 months, that is since (date), because of your sexual orientation?”
- Lifetime history of being bullied, using a binary measure based on responses to questions in the Stressful Life Events section of APMS. The wording for this item was based on the List of Threatening Life Experiences (LTE) [29].

### Putative moderators

To test for evidence that associations might differ by (i) gender and (ii) survey year (to ascertain whether inequalities had persisted between 2007 and 2014), we conducted interaction tests for these variables.

### Statistical analysis

We compared the proportions of those self-identifying as each sexual orientation group for the years 2007 and 2014 and overall. We used descriptive statistics to describe the socio-demographic and clinical characteristics of our sample by sexual orientation group (combining 2007 and 2014 data), testing bivariate associations. Using the combined 2007/2014 survey samples, we ran univariable logistic regression models (model 1) to examine the association of sexual orientation with past-year suicidal thoughts, past-year suicide attempt and lifetime non-suicidal self-harm**.** Our multivariable logistic regression models used successive block adjustments: in model 2, we adjusted for socio-demographic covariates (age, gender, educational attainment, IMD quintile) and sample year; in model 3 (the fully adjusted model) we adjusted for socio-demographic variables, survey year, and common mental disorder (CMD).

To test whether specific minority stress variables attenuated the main associations between sexual orientation and suicidality, we added to our final models (separately), past-year discrimination (model 4) and lifetime history of being bullied (model 5) to generate hypotheses about potential mediating roles.

We tested for effect modification of (i) gender and (ii) survey year on fully adjusted associations (model 3), by adding these as interaction terms to final models.

To gain a sense of longer-term patterns of suicidality over the course of sexual identity milestones, we added a post hoc analysis investigating the associations between sexual orientation and lifetime suicidal thoughts and suicide attempt.

All analyses were conducted using data weighted to take account of the complex survey design and of non-response. This ensured that our estimates were representative of the household population in England. For the 2007 survey, we used new weightings as provided by APMS in 2018. This involved use of the relevant survey (*svy*) commands in Stata 15, which allow for the use of clustered data modified by probability weights and provide robust estimates of variance.

## Results

### Sexual orientation characteristics

Our dataset included 10,443 people aged 16–64 years; 5,386 from the 2007 survey and 5,057 from the 2014 survey (Table 1). For 2007, the largest sexual orientation group was that identifying as heterosexual and mainly heterosexual (96.3%), followed by those identifying as other (1.8%), lesbian/gay or mainly homosexual (1.2%), and bisexual (0.8%). Responses from the 2014 survey identified a slightly smaller proportion identifying as heterosexual and mainly heterosexual (95.6%), slightly higher proportions identifying as lesbian/gay or mainly homosexual (1.5%) and as bisexual (1.5%) and a slightly lower proportion identifying as other (1.2%). Combined responses for both years showed that individuals defined their sexuality as: heterosexual (96.0%), bisexual (1.1%), lesbian/gay (1.5%) and other (1.5%).

**Table 1:** Proportions of those self-defining sexual orientation overall and by survey year

	2007		2014		Combined Total	
Sexual orientation	*n*	% (95% CI)	*n*	% (95% CI)	*n*	% (95% CI)
Heterosexual and mainly heterosexual	5,182	96.3 [95.7, 96.8]	4834	95.6 [94.9, 96.2]	10,016	96.0 [95.5, 96.4]
Bisexual	42	0.8 [0.6, 1.1]	74	1.5 [1.1, 1.9]	116	1.1 [0.9, 1.4]
Lesbian/gay and mainly homosexual	70	1.2 [0.9, 1.6]	93	1.8 [1.4, 2.2]	163	1.5 [1.2, 1.8]
Other	92	1.8 [1.4, 2.2]	56	1.2 [0.9, 1.6]	148	1.5 [1.2, 1.8]
Total	5,386			5,057		10,443

### Socio-demographic and clinical characteristics

Weighted estimates of the socio-demographic and clinical characteristics of the sample (generalisable to the English population) show that, overall, the group identifying as bisexual represented a low proportion of men (27.5%), whilst men represented the majority gender in the group identifying as lesbian/gay (69.6%; Table 2). The bisexual group had the lowest mean age (33.4; standard deviation = 1.4). The highest proportion of those identifying as white were in the bisexual group (92.0%), the highest proportion educated to degree level were those who identified as lesbian/gay (35.9%), and the highest proportion in the lowest-deprivation quintile were in the heterosexual group (18.9%).

**Table 2:** Socio-demographic and clinical characteristics by self-defined sexual orientation

Characteristic	Heterosexual	Bisexual	Lesbian/Gay	Other	*p* value
Gender (male) %	49.9	27.5	69.6	44.9	< 0.001
Age (years) mean (SE)	39.7 (0.2)	33.4 (1.4)	38.1 (1.2)	38.2 (1.4)	< 0.001
White ethnicity %	87.3	92.0	89.4	61.4	< 0.001
Qualification %					
Degree	26.1	22.9	35.9	13.4	< 0.001
Teaching, HND, nursing	7.8	4.6	9.2	3.2	
A level	19.8	21.3	20.3	6.8	
GCSE or equivalent	29.4	35.6	26.5	35.1	
Foreign/other qualification	2.1	1.2	0.7	11.7	
No qualification	14.9	14.5	7.4	29.9	
Area-level deprivation (IMD quintiles) %					0.011
1 (least deprived)	18.9	14.1	10.8	14.1	
2	20.9	17.1	21.7	14.3	
3	19.7	15.6	16.2	15.4	
4	19.9	27.3	26.1	25.8	
5 (most deprived)	20.6	25.9	25.2	30.5	
Minority stress variables %					
Past-year discrimination based on sexual orientation	0.3	9.4	23.2	2.7	< 0.001
Lifetime history of being bullied	25.3	47.5	51.7	22.9	< 0.001
Suicidality and self-harm %					
Suicidal thoughts, past-week	0.9	2.0	1.0	1.5	0.483
Suicidal thoughts, past-year	5.0	13.4	11.4	8.8	< 0.001
Suicidal thoughts, lifetime	16.7	47.0	36.7	18.4	< 0.001
Suicide attempt, past-week	< 1	0.0	0.0	0.0	0.990
Suicide attempt, past-year	0.7	4.3	1.1	1.5	< 0.001
Suicide attempt, lifetime	5.4	24.1	15.2	8.9	< 0.001
Non-suicidal self-harm, lifetime	5.3	26.9	15.2	9.4	< 0.001

Table does not show missing data as weighted estimates are presented, but overall there was a low level of missing data

*SE* standard error; *GCSE* General Certificate of Secondary Education, typically taken at age 16; *HND* Higher National Diploma; *IMD* Index of Multiple Deprivation

The group who identified as lesbian/gay had the highest prevalence of lifetime bullying victimisation (51.7%) and of past-year discrimination due to sexual orientation (23.2%).

For all suicidality and self-harm outcomes, prevalence was lowest in the group identifying as heterosexual (apart from past-week suicide attempt where there were no differences). Thus, the prevalence of past-year suicidal thoughts was lowest in the group identifying as heterosexual (5.0%) compared with those identifying as bisexual (13.4%), lesbian/gay (11.4%) or other (8.8%). The prevalence of past-year suicide attempt was lowest in the group identifying as heterosexual (0.7%) compared with those identifying as bisexual (4.3%), lesbian/gay (1.1%) or as other (1.58%). The prevalence of lifetime non-suicidal self-harm was lowest in the group identifying as heterosexual (5.3%) compared with those identifying as bisexual (26.9%), lesbian/gay (15.2%) or other (9.4%).

### Associations between sexual orientation and suicidal/self-harm outcomes

#### Associations between sexuality and past-year suicidal thoughts

Our unadjusted model (model 1) showed that those who identified as bisexual and as lesbian/gay were significantly more likely to report past-year suicidal thoughts than heterosexuals (Table 3; Supplementary Table 1), remaining significant when adjusted for socio-demographic factors. However, in the fully adjusted model, these associations remained significant only for those who identified as lesbian/gay, albeit attenuated.

**Table 3:**
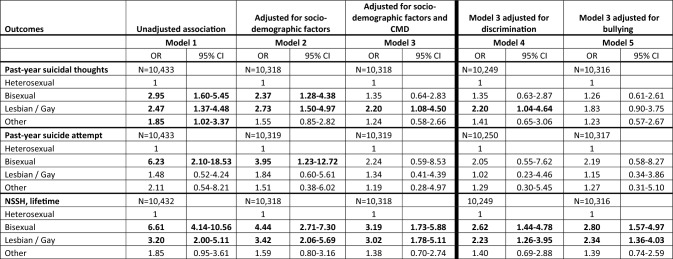
Associations between sexual orientation and suicidality and self-harm outcomes

Bold values denote that estimates were significant at a *p* value threshold of 0.05

*CI* confidence interval; *CMD* common mental disorder; *NSSH* non-suicidal self-harm; *OR* odds ratio

This estimate for the lesbian/gay group remained unchanged after further addition of discrimination (model 4) but was attenuated and became non-significant when separately adding bullying (model 5).

#### Associations between sexuality and past-year suicide attempt

Our unadjusted model showed that those who identified as bisexual (but no other sexual minority group) were more likely to report past-year suicide attempt than heterosexuals. Whilst this remained significant in a model adjusted for socio-demographic factors, it was no longer significant in a fully adjusted model taking into account CMD (Table 3; Supplementary Table 2).

Further addition of discrimination (model 4) and of bullying (model 5) each attenuated the odds ratio estimate for the bisexual group marginally, but both associations remained non-significant.

#### Associations between sexuality and lifetime non-suicidal self-harm

In unadjusted and fully adjusted models, the groups identifying as bisexual and as lesbian/gay were each significantly more likely to report lifetime non-suicidal self-harm than heterosexuals, with some attenuation when taking into account CMD (Table 3; Supplementary Table 3). No such association was observed for the group identifying as other.

Adding discrimination (model 4) and bullying (model 5) to final models for the groups identifying as bisexual and as lesbian/gay attenuated the associations slightly in each case, but each remained significant.

#### Post hoc analyses of lifetime measures

To set the above findings in a longer-term context, we found that people identifying as bisexual and as lesbian/gay were significantly more likely to report lifetime suicidal thoughts and lifetime suicide attempt than heterosexuals in adjusted analyses (Supplementary Tables 4 and 5).

#### Interactions with gender and with survey year

Our interaction tests found no evidence to support effect modification by gender or by survey year (2007 vs. 2014) for any of our final models (Table 4; Supplementary Tables 6 and 7).

**Table 4:** Interaction tests for effect modification by survey year and gender

	Past-year suicidal thoughts	Past-year suicide attempts	NSSH, lifetime
	*p* value	*p* value	*p* value
*Interaction with gender*			
Bisexual*male	0.651	0.517	0.357
Lesbian/gay*male	0.805	0.285	0.102
Other*male	0.128	0.105	0.500
*Interaction with survey year*			
Bisexual*2014	0.792	0.145	0.204
Lesbian/gay*2014	0.358	0.573	0.474
Other*2014	0.550	0.355	0.286

Threshold of *p* ≥ 0.05 for all tests

*NSSH* non-suicidal self-harm

## Discussion

### Main findings

Our analysis of English household survey data from 2007 and 2014 found that, compared with heterosexuals, the prevalence of past-year suicidal thoughts was significantly elevated in lesbian/gay individuals, and the prevalence of lifetime non-suicidal self-harm was significantly elevated in both lesbian/gay and bisexual individuals. However, we found no elevated risk of past-year suicide attempt in any individual sexual minority group compared with heterosexuals once accounting for CMD. Stepwise adjustments suggested that CMD accounted for a component of the elevated risk for suicidal ideation and attempt observed in partially adjusted models for individuals identifying as bisexual. However, this would require formal testing using mediation analysis.

Our descriptive findings demonstrated a much higher prevalence of discrimination on grounds of sexual orientation in the lesbian/gay group relative to other groups, and (to a lesser extent) of lifetime bullying, but also a high prevalence of each in the bisexual group. There was some evidence to support a role for each of these minority stress variables in the association between sexual minority status and lifetime non-suicidal self-harm, in that each attenuated slightly the associations for lesbian/gay and bisexual individuals. There was some evidence to support bullying as contributing to the association between lesbian/gay identity and past-year suicidal thoughts, but no evidence to support discrimination in this role. These findings are hypothesis generating regarding signals of potential mediators, and indicate a need for formal mediation analysis using longitudinal datasets.

One explanation for the relatively greater attenuating effect of discrimination and bullying in models for the lesbian/gay group than the bisexual group in relation to non-suicidal self-harm was that, in this sample, they were more likely to be older than other groups (whilst the bisexual group were more likely to be younger and female). The lesbian/gay group may have accumulated more toxic or memorable experiences of bullying or discrimination over years of limited or no legal protections [30], coping with this through self-harm. Another explanation is that bisexual individuals may be less visible, with evidence that they are more likely to keep their identity private to avoid discrimination [31], potentially rendering them less susceptible to the effect of discrimination or bullying.

Generally, these group differences reinforce the importance of disaggregating sexual minority groups when investigating self-harm and suicidality outcomes rather than crude comparisons of heterosexual vs. non-heterosexual groups. We were also able to test whether associations differed by gender, finding no evidence to support this. Finally, we found no evidence that disparities in past-year suicidal thoughts or lifetime non-suicidal self-harm differed in 2007 and 2014. This is despite an apparent liberalisation in societal views about same-sex relationships over this period [12].

## Findings in the context of other evidence

Our findings regarding the absence of an association with suicide attempt, but a clear association with non-suicidal self-harm, are consistent with those of a previous UK schools-based survey [16]. However, more generally, our findings go beyond those reported in previous systematic reviews because of our ability to report comparisons between specific sexual minority groups and separate out specific suicidality outcomes. Whilst a previous meta-analysis found an elevated risk of suicidality in lesbian women and in gay men [17], this did not separate outcomes into suicidal ideation, suicide attempt and self-harm. Our own study found specific associations of lesbian/gay identity with suicidal thoughts, and of lesbian/gay and bisexual identity with non-suicidal self-harm. It is possible, given the limited statistical power of interaction tests, that our finding of no effect modification by gender may reflect inadequate sample size given our use of tighter outcomes.

The striking excess risk of lifetime non-suicidal self-harm amongst bisexual individuals is consistent with findings from previous reviews focussed on young people [15] and individuals in all age groups [17]. More work is needed to understand why bisexual people appear more likely than lesbian/gay people to cope with distress by self-harming. Our findings regarding signals of mediation by minority stress variables in the associations with suicidal ideation and non-suicidal self-harm add to evidence that discrimination and bullying on grounds of sexuality (including within the home) may help explain disparities in suicide-related outcomes [9, 10, 15]. Another potential contributor suggested by the literature on mental health disparities in sexual minorities is stigma [23]. Although we lacked a stigma variable, it is possible that stigma and discrimination are implicated in the development of common mental disorders, and that these in turn increase risk of self-harm and suicidality. Further work is needed to test the mechanistic hypotheses generated here using longitudinal data, informing the development of interventions that might address stigma, discrimination, bullying and other putative mechanistic factors.

Our prevalence estimates for lesbian/gay (1.5%), bisexual (1.1%), and other (1.5%) sexual orientations in an English household sample, with 4.1% overall defined as a sexual minority, are slightly lower than those from other studies using probabilistic sampling. Findings from a 2010 British household survey estimated that 2.5% of men and 2·4% of women self-identified as LGB, with higher estimates for same-sex attraction (6.5% and 11.5%, respectively) and same-sex sexual activity (5.5% and 6.1%, respectively) [32]. In a UK birth cohort, 1.7% of 13 year-olds self-identified as exclusively bisexual, 0.3% self-identified as exclusively gay, with 12.6% overall categorised as a sexual minority [13]. This is greater than the 3.2% estimated for the whole population in the 2021 England and Wales census [5], and is likely due to reduced stigma and greater fluidity of sexual identity in younger people [13]. Estimates based on same-sex marriages registered in Denmark and Sweden over the period 1989–2016 were 0.7% [3]. A small population-based study of Dutch adults defined by recent sexual activity identified a prevalence of 2.8% for men and 1.4% for women of same-sex partners, but this was only amongst those who were sexually active [9]. Precision in the dimensions of sexual orientation measured are clearly important when interpreting public health implications [32].

## Strengths and limitations

We analysed data on a large probability sample representative of England households, being the first population-based sample in the UK investigating suicidal thoughts, suicide attempt and non-suicidal self-harm by sexual orientation group. Combining data from two surveys meant that we had sufficient power to compare specific sexual minority groups, identifying important disparities within minority groups as well as more broadly when compared to heterosexual controls. Use of a large representative sample, narrow validated measures and carefully selected confounders meant that this study overcomes many of the methodological limitations of previous studies conducted in a British sample. Computer-assisted data collection for sensitive questions is also likely to have enhanced disclosure on our main exposure and outcome measures, reducing the potential for social desirability bias. Use of weightings ensured that our sample was representative of English households, although findings may not be generalisable to other UK devolved nations or countries outside the UK due to cultural differences. Due to the low level of missing data on key variables, all regression models involved samples of over 10,000 individuals. Although use of data from surveys at two separate time points allowed us to test for changes in disparities over a 7-year period, a longitudinal cohort would have been optimal, sampling the same individuals. Given the cross-sectional design, we could not be certain that the minority stress variables selected as putative mediators preceded outcomes. However, we assumed these might capture a trajectory of discriminatory or bullying experiences preceding suicidality or self-harm. The 2007 and 2014 APMS surveys did not distinguish birth sex and gender identity, so the measure used in this study lacks validity. Other limitations of this study are the lack of data on people over the age of 64, and the inclusion of the 22 individuals in 2007 who described themselves as “mainly heterosexual” within the “heterosexual and mainly heterosexual” group (despite their potentially higher risk of suicidality relating to their sexual identity). Future waves of data collection for the Adult Psychiatric Morbidity Surveys (from 2022) will collect detailed data on gender identity and sexuality in all age groups.

## Clinical, policy and research recommendations

These findings extend our understanding of the excess risk of self-harm and suicidality in non-heterosexuals by comparing specific outcomes in specific sexual minority groups. They highlight clearly that non-suicidal self-harm is a clinical issue for non-heterosexuals across the life course, that suicidal thoughts are particularly pronounced for bisexual individuals, and that common mental disorder and experiences of victimisation may contribute to self-harm and suicidal thinking. Clinicians should be aware of these issues when caring for patients from sexual minority groups, probing sensitively for experiences of discrimination or bullying and signs of CMD, as part of screening for suicidality. Such factors will help in therapeutic assessment and management of risk [33], taking into account the historical and dynamic risk factors that interplay in precipitating suicidal ideation and suicide attempt [34]. Risk management strategies might also include challenging the cognitive biases engendered through minority stress factors, and measures to counter everyday bullying and discrimination. Society has an important role to play in this too. Government departments (such as the UK Government Equalities Office), educational settings, workplaces, and individuals need to consider their own cultures and attitudes towards people from sexual minorities when reviewing our finding of no narrowing in suicidality outcomes over the period 2017–2014.

It is possible that the specific health inequalities we reported relate to systemic issues within health service provision, especially in geographical locations or clinical services less accepting of LGB groups. We lacked the spatial variables to investigate this. Patients perceiving heteronormative environments may be less likely to disclose their sexuality, for fear of discrimination, and this hampers an understanding of their health and social needs. Services seeking to address insidious inequalities could consider raising awareness of research findings such as those presented here, recruitment of healthcare staff representing different sexual and gender minority groups, and staff training to recognise and limit heteronormative bias, including therapists [35]. These measures may help encourage disclosure of sexuality and of suicidality, promoting therapeutic alliance, and improving our responses to suicidal thoughts and self-harm in LGB groups. Providing community-based support to LGB groups is also essential, complementing appropriate healthcare provision, but is reliant on sustained funding for such voluntary sector organisations.

Further longitudinal research is needed to understand the trajectories of self-harm and suicidality in specific LGB groups and associations with mental health. Formal mediation analyses are needed to understand the roles of victimisation, family environment, stigma and other putative mediators. More work is needed to understand disparities in self-harm and suicidality amongst older LGB adults, who were not investigated in this study. This is important because of an ageing population, this group’s long experience of hostile social attitudes [30] and the importance of risk factors such as isolation in older adults [36]. The collection of sexuality data in routine administrative, clinical and survey-based datasets is essential in surveillance of suicidality in LGB people. Routine monitoring of sexuality in mental health services became compulsory in the UK in 2020 [37], yet UK mortality statistics are not broken down by sexuality, detailed gender categories or ethnicity. Such data are required to allow exploration of intersectional differences. Across a range of datasets, such work is needed to understand the needs of LGB groups who identify as transgender, migrants or specific ethnic minority groups. In all such studies, it is important to avoid aggregating sexual minority groups because of intra-group differences we have demonstrated in their mental health [23], self-harm and suicidality. Qualitative research will also help identify putative mediators of these associations in specific groups, as well as the acceptability of suggested interventions.

## Conclusion

Our findings from a representative household sample in England identify a heightened risk of past-year suicidal thinking and lifetime non-suicidal self-harm amongst people who identify as bisexual or as lesbian/gay compared with heterosexuals. There was an evident contribution of common mental disorder and of discrimination and bullying, and findings were similar for individuals in both gender categories captured. These disparities had not narrowed over the period spanning 2007–2014, despite the range of measures taken to protect the rights of LGB people. More research is needed to understand the mechanisms underlying these associations, why bullying and discrimination due to sexual orientation is so prevalent in lesbian/gay and bisexual people, how society can prevent the emergence of suicidal thoughts and self-harm in LGB groups and how health services might better meet their needs.

### Supplementary Information

Below is the link to the electronic supplementary material.

Supplementary file1 (DOCX 62 KB)

## Data Availability

Access to the 2007 APMS dataset is via the UK Data Service: https://beta.ukdataservice.ac.uk/datacatalogue/studies/study?id=6379. Access to the 2014 APMS dataset is by formal application to NHS Digital.
