# Supplementary material for: Suicidal thoughts, suicide attempt and non-suicidal self-harm amongst lesbian, gay and bisexual adults compared with heterosexual adults: analysis of data from two nationally representative English household surveys
Source: Soc Psychiatry Psychiatr Epidemiol. 2023 Jun 9;59(2):273–83. doi: 10.1007/s00127-023-02490-4 (PMC10838834; doi:10.1007/s00127-023-02490-4)
Supplement: Supplementary file 1 — Supplementary file1 (DOCX 62 KB) [file 127_2023_2490_MOESM1_ESM.docx]

**Supplementary Table 1: Associations between sexual orientation and past-year suicidal thoughts**

|  | **Unadjusted association** | | **Adjusted for socio-demographic factors** | | **Adjusted for socio-demographic factors and CMD** | | **Model 3 adjusted for discrimination** | | **Model 3 adjusted for bullying** | |
| --- | --- | --- | --- | --- | --- | --- | --- | --- | --- | --- |
|  | **Model 1** | | **Model 2** | | **Model 3 (final model)** | | **Model 4** | | **Model 5** | |
|  | OR | 95% CI | OR | 95% CI | OR | 95% CI | OR | 95% CI | OR | 95% CI |
| **Past-year suicidal thoughts** | N=10,433 |  | N=10,318 |  | N=10,318 |  | N=10,249 |  | N=10,316 |  |
| **Sexuality** |  |  |  |  |  |  |  |  |  |  |
| Heterosexual | 1 |  | 1 |  | 1 |  | 1 |  | 1 |  |
| Bisexual | **2.95** | **1.60-5.45** | **2.37** | **1.28-4.38** | 1.35 | 0.64-2.83 | 1.35 | 0.63-2.87 | 1.26 | 0.61-2.61 |
| Lesbian / Gay | **2.47** | **1.37-4.48** | **2.73** | **1.50-4.97** | **2.20** | **1.08-4.50** | **2.20** | **1.04-4.64** | 1.83 | 0.90-3.75 |
| Other | **1.85** | **1.02-3.37** | 1.55 | 0.85-2.82 | 1.24 | 0.58-2.66 | 1.41 | 0.65-3.06 | 1.23 | 0.57-2.67 |
|  |  |  |  |  |  |  |  |  |  |  |
| Year 2007 |  |  | 1 |  | 1 |  | 1 |  | 1 |  |
| Year 2014 |  |  | 1.31 | 1.07-1.60 | 1.27 | 1.01-1.58 | 1.30 | 1.05-1.63 | 1.24 | 1.00-1.56 |
|  |  |  |  |  |  |  |  |  |  |  |
| Male gender |  |  | 0.76 | 0.62-0.93 | 1.04 | 0.84-1.31 | 1.04 | 0.83-1.31 | 1.07 | 0.85-1.34 |
| Age |  |  | 0.98 | 0.98-0.99 | 0.98 | 0.97-0.99 | 0.98 | 0.97-0.99 | 0.98 | 0.98-0.99 |
|  |  |  |  |  |  |  |  |  |  |  |
| Qualifications |  |  |  |  |  |  |  |  |  |  |
| Degree |  |  | 1 |  | 1 |  | 1 |  | 1 |  |
| Teaching, HND, Nursing |  |  | 0.93 | 0.59-1.48 | 0.83 | 0.51-1.36 | 0.85 | 0.52-1.39 | 0.82 | 0.50-1.35 |
| A level |  |  | 1.00 | 0.70-1.43 | 0.85 | 0.59-1.22 | 0.84 | 0.59-1.21 | 0.84 | 0.58-1.22 |
| GCSE or equivalent |  |  | 1.60 | 1.20-2.14 | 1.28 | 0.94-1.73 | 1.29 | 0.95-1.74 | 1.29 | 0.95-1.76 |
| Foreign/other qualification |  |  | 1.07 | 0.53-2.18 | 1.05 | 0.49-2.26 | 1.09 | 0.50-2.36 | 1.09 | 0.49-2.45 |
| No qualification |  |  | 2.22 | 1.64-3.02 | 1.67 | 1.21-2.30 | 1.71 | 1.23-2.36 | 1.78 | 1.29-2.46 |
|  |  |  |  |  |  |  |  |  |  |  |
| Area-level deprivation (IMD quintiles) |  |  |  |  |  |  |  |  |  |  |
| 1 (least deprived) |  |  | 1 |  | 1 |  | 1 |  | 1 |  |
| 2 |  |  | 1.45 | 1.02-2.07 | 1.31 | 0.89-1.92 | 1.30 | 0.89-1.91 | 1.30 | 0.88-1.92 |
| 3 |  |  | 1.85 | 1.29-2.68 | 1.43 | 0.97-2.12 | 1.39 | 0.94-2.06 | 1.48 | 0.99-2.21 |
| 4 |  |  | 1.63 | 1.15-2.31 | 1.26 | 0.87-1.83 | 1.24 | 0.85-1.79 | 1.29 | 0.89-1.87 |
| 5 (most deprived) |  |  | 2.12 | 1.49-3.03 | 1.45 | 0.99-2.13 | 1.42 | 0.96-2.09 | 1.48 | 1.01-2.19 |
|  |  |  |  |  |  |  |  |  |  |  |
| Common mental disorder |  |  |  |  | 13.80 | 10.96-17.38 | 13.88 | 11.06-17.42 | 12.09 | 9.52-15.35 |
| Past-year discrimination on grounds of sexual orientation |  |  |  |  |  |  | 1.03 | 0.41-2.56 |  |  |
| Lifetime history of being bullied |  |  |  |  |  |  |  |  | 2.08 | 1.65-2.63 |

**Supplementary Table 2: Associations between sexual orientation and past-year suicide attempt**

|  | **Unadjusted association** | | **Adjusted for socio-demographic factors** | | **Adjusted for socio-demographic factors and CMD** | | **Model 3 adjusted for discrimination** | | **Model 3 adjusted for bullying** | |
| --- | --- | --- | --- | --- | --- | --- | --- | --- | --- | --- |
|  | **Model 1** | | **Model 2** | | **Model 3 (final model)** | | **Model 4** | | **Model 5** | |
|  | OR | 95% CI | OR | 95% CI | OR | 95% CI | OR | 95% CI | OR | 95% CI |
| **Past-year suicide attempt** | N=10,433 |  | N=10,319 |  | N=10,319 |  | N=10,250 |  | N=10,317 |  |
| **Sexuality** |  |  |  |  |  |  |  |  |  |  |
| Heterosexual | 1 |  | 1 |  | 1 |  | 1 |  | 1 |  |
| Bisexual | **6.23** | **2.10-18.53** | **3.95** | **1.23-12.72** | 2.24 | 0.59-8.53 | 2.05 | 0.55-7.62 | 2.19 | 0.58-8.27 |
| Lesbian / Gay | 1.48 | 0.52-4.24 | 1.84 | 0.60-5.61 | 1.34 | 0.41-4.39 | 1.02 | 0.23-4.46 | 1.15 | 0.34-3.86 |
| Other | 2.11 | 0.54-8.21 | 1.51 | 0.38-6.02 | 1.19 | 0.28-4.97 | 1.29 | 0.30-5.45 | 1.27 | 0.31-5.10 |
|  |  |  |  |  |  |  |  |  |  |  |
| Year 2007 |  |  | 1 |  | 1 |  | 1 |  | 1 |  |
| Year 2014 |  |  | 1.15 | 0.67-1.97 | 1.09 | 0.62-1.91 | 1.10 | 0.63-1.93 | 1.08 | 0.61-1.90 |
|  |  |  |  |  |  |  |  |  |  |  |
| Male gender |  |  | 0.66 | 0.38-1.15 | 0.99 | 0.55-1.76 | 0.99 | 0.55-1.77 | 1.01 | 0.56-1.82 |
| Age |  |  | 0.96 | 0.94-0.98 | 0.95 | 0.93-0.97 | 0.95 | 0.93-0.97 | 0.96 | 0.94-0.98 |
|  |  |  |  |  |  |  |  |  |  |  |
| Qualifications |  |  |  |  |  |  |  |  |  |  |
| Degree |  |  | 1 |  | 1 |  | 1 |  | 1 |  |
| Teaching, HND, Nursing |  |  | 1.13 | 0.32-3.95 | 0.99 | 0.28-3.54 | 0.97 | 0.27-3.46 | 1.00 | 0.28-3.58 |
| A level |  |  | 1.29 | 0.43-3.85 | 1.10 | 0.37-3.31 | 1.08 | 0.36-3.25 | 1.09 | 0.37-3.28 |
| GCSE or equivalent |  |  | 3.60 | 1.67-7.78 | 2.71 | 1.24-5.94 | 2.68 | 1.23-5.86 | 2.75 | 1.25-6.03 |
| Foreign/other qualification |  |  | 1.01 | 0.12-8.36 | 0.99 | 0.12-8.26 | 0.99 | 0.12-8.23 | 1.12 | 0.13-9.38 |
| No qualification |  |  | 6.24 | 2.55-15.28 | 4.52 | 1.83-11.18 | 4.46 | 1.80-11.04 | 4.75 | 1.93-11.68 |
|  |  |  |  |  |  |  |  |  |  |  |
| Area-level deprivation (IMD quintiles) |  |  |  |  |  |  |  |  |  |  |
| 1 (least deprived) |  |  | 1 |  | 1 |  | 1 |  | 1 |  |
| 2 |  |  | 0.96 | 0.33-2.78 | 0.84 | 0.29-2.48 | 0.86 | 0.29-2.50 | 0.85 | 0.29-2.54 |
| 3 |  |  | 1.22 | 0.46-3.24 | 0.88 | 0.34-2.29 | 0.90 | 0.35-2.34 | 0.94 | 0.36-2.44 |
| 4 |  |  | 0.84 | 0.31-2.28 | 0.65 | 0.23-1.79 | 0.67 | 0.24-1.84 | 0.68 | 0.25-1.89 |
| 5 (most deprived) |  |  | 1.18 | 0.47-2.99 | 0.82 | 0.33-2.03 | 0.85 | 0.34-2.10 | 0.86 | 0.35-2.15 |
|  |  |  |  |  |  |  |  |  |  |  |
| Common mental disorder |  |  |  |  | 18.67 | 9.01-38.70 | 18.58 | 8.99-38.43 | 16.37 | 7.64-35.07 |
| Past-year discrimination on grounds of sexual orientation |  |  |  |  |  |  | 1.74 | 0.41-7.30 |  |  |
| Lifetime history of being bullied |  |  |  |  |  |  |  |  | 1.86 | 1.04-3.33 |

**Supplementary Table 3: Associations between sexual orientation and lifetime NSSH**

|  | **Unadjusted association** | | **Adjusted for socio-demographic factors** | | **Adjusted for socio-demographic factors and CMD** | | **Model 3 adjusted for discrimination** | | **Model 3 adjusted for bullying** | |
| --- | --- | --- | --- | --- | --- | --- | --- | --- | --- | --- |
|  | **Model 1** | | **Model 2** | | **Model 3 (final model)** | | **Model 4** | | **Model 5** | |
|  | OR | 95% CI | OR | 95% CI | OR | 95% CI | OR | 95% CI | OR | 95% CI |
| **Lifetime NSSH** | N=10,432 |  | N=10,318 |  | N=10,318 |  | 10,249 |  | N=10,316 |  |
| **Sexuality** |  |  |  |  |  |  |  |  |  |  |
| Heterosexual | 1 |  | 1 |  | 1 |  | 1 |  | 1 |  |
| Bisexual | **6.61** | **4.14-10.56** | **4.44** | **2.71-7.30** | **3.19** | **1.73-5.88** | **2.62** | **1.44-4.78** | **2.80** | **1.57-4.97** |
| Lesbian / Gay | **3.20** | **2.00-5.11** | **3.42** | **2.06-5.69** | **3.02** | **1.78-5.11** | **2.23** | **1.26-3.95** | **2.34** | **1.36-4.03** |
| Other | 1.85 | 0.95-3.61 | 1.59 | 0.80-3.16 | 1.38 | 0.70-2.74 | 1.40 | 0.69-2.88 | 1.39 | 0.74-2.59 |
|  |  |  |  |  |  |  |  |  |  |  |
| Year 2007 |  |  | 1 |  | 1 |  | 1 |  | 1 |  |
| Year 2014 |  |  | 1.82 | 1.50-2.21 | 1.81 | 1.48-2.20 | 1.79 | 1.47-2.18 | 1.79 | 1.46-2.19 |
|  |  |  |  |  |  |  |  |  |  |  |
| Male gender |  |  | 0.71 | 0.57-0.87 | 0.87 | 0.70-1.08 | 0.88 | 0.70-1.09 | 0.88 | 0.70-1.10 |
| Age |  |  | 0.95 | 0.95-0.96 | 0.95 | 0.94-0.96 | 0.95 | 0.94-0.96 | 0.95 | 0.94-0.96 |
|  |  |  |  |  |  |  |  |  |  |  |
| Qualifications |  |  |  |  |  |  |  |  |  |  |
| Degree |  |  | 1 |  | 1 |  | 1 |  | 1 |  |
| Teaching, HND, Nursing |  |  | 0.76 | 0.49-1.20 | 0.69 | 0.44-1.10 | 0.69 | 0.43-1.10 | 0.69 | 0.44-1.09 |
| A level |  |  | 0.84 | 0.61-1.16 | 0.76 | 0.54-1.06 | 0.73 | 0.52-1.03 | 0.74 | 0.53-1.04 |
| GCSE or equivalent |  |  | 1.21 | 0.92-1.59 | 1.04 | 0.79-1.37 | 1.02 | 0.77-1.35 | 1.05 | 0.79-1.39 |
| Foreign/other qualification |  |  | 0.75 | 0.35-1.61 | 0.77 | 0.37-1.60 | 0.80 | 0.39-1.67 | 0.92 | 0.44-1.93 |
| No qualification |  |  | 1.61 | 1.16-2.22 | 1.34 | 0.97-1.85 | 1.29 | 0.94-1.76 | 1.48 | 1.07-2.06 |
|  |  |  |  |  |  |  |  |  |  |  |
| Area-level deprivation (IMD quintiles) |  |  |  |  |  |  |  |  |  |  |
| 1 (least deprived) |  |  | 1 |  | 1 |  | 1 |  | 1 |  |
| 2 |  |  | 1.18 | 0.83-1.68 | 1.13 | 0.78-1.64 | 1.14 | 0.79-1.66 | 1.13 | 0.77-1.66 |
| 3 |  |  | 1.52 | 1.07-2.16 | 1.32 | 0.92-1.90 | 1.32 | 0.92-1.90 | 1.41 | 0.97-2.04 |
| 4 |  |  | 1.44 | 1.02-2.04 | 1.26 | 0.88-1.81 | 1.21 | 0.84-1.73 | 1.31 | 0.90-1.89 |
| 5 (most deprived) |  |  | 2.01 | 1.44-2.82 | 1.65 | 1.16-2.33 | 1.69 | 1.19-2.39 | 1.76 | 1.23-2.51 |
|  |  |  |  |  |  |  |  |  |  |  |
| Common mental disorder |  |  |  |  | 5.59 | 4.51-6.91 | 5.64 | 4.56-6.99 | 4.50 | 3.60-5.62 |
| Past-year discrimination on grounds of sexual orientation |  |  |  |  |  |  | 2.50 | 1.17-5.35 |  |  |
| Lifetime history of being bullied |  |  |  |  |  |  |  |  | 3.30 | 2.65-4.12 |

**Supplementary Table 4: Associations between sexual orientation and lifetime suicidal thoughts (*post hoc* analysis)**

|  | **Unadjusted association** | | **Adjusted for socio-demographic factors** | | **Adjusted for socio-demographic factors and CMD** | | **Model 3 adjusted for discrimination** | | **Model 3 adjusted for bullying** | |
| --- | --- | --- | --- | --- | --- | --- | --- | --- | --- | --- |
|  | **Model 1** | | **Model 2** | | **Model 3 (final model)** | | **Model 4** | | **Model 5** | |
|  | OR | 95% CI | OR | 95% CI | OR | 95% CI | OR | 95% CI | OR | 95% CI |
| **Lifetime suicidal thoughts** | N=10,433 |  | N=10,318 |  | N=10,318 |  | 10,249 |  | N=10,316 |  |
| **Sexuality** |  |  |  |  |  |  |  |  |  |  |
| Heterosexual | 1 |  | 1 |  | 1 |  | 1 |  | 1 |  |
| Bisexual | **4.43** | **2.92-6.7** | **3.94** | **2.57-6.02** | **3.15** | **2.05-4.84** | **3.06** | **1.98-4.75** | **2.76** | **1.76-4.32** |
| Lesbian / Gay | **2.90** | **2.00-4.20** | **3.10** | **2.14-4.5** | **2.92** | **1.93-4.42** | **2.52** | **1.55-4.077** | **2.30** | **1.51-3.51** |
| Other | 1.13 | 0.73-1.76 | 1.08 | 0.68-1.70 | 0.91 | 0.51-1.63 | 0.97 | 0.54-1.75 | 0.93 | 0.51-1.67 |
|  |  |  |  |  |  |  |  |  |  |  |
| Year 2007 |  |  | 1 |  | 1 |  | 1 |  | 1 |  |
| Year 2014 |  |  | 1.35 | 1.19-1.52 | 1.35 | 1.18-1.54 | 1.35 | 1.19-1.55 | 1.30 | 1.14-1.49 |
|  |  |  |  |  |  |  |  |  |  |  |
| Male gender |  |  | 0.76 | 0.68-0.86 | 0.92 | 0.81-1.05 | 0.92 | 0.81-1.04 | 0.92 | 0.81-1.05 |
| Age |  |  | 1 | 0.99-1 | 1 | 0.99-1 | 1 | 0.99-1 | 1 | 1-1.01 |
|  |  |  |  |  |  |  |  |  |  |  |
| Qualifications |  |  |  |  |  |  |  |  |  |  |
| Degree |  |  | 1 |  | 1 |  | 1 |  | 1 |  |
| Teaching, HND, Nursing |  |  | 1.03 | 0.80-1.33 | 0.96 | 0.73-1.26 | 0.97 | 0.74-1.28 | 0.96 | 0.74-1.26 |
| A level |  |  | 1.25 | 1.04-1.50 | 1.16 | 0.96-1.41 | 1.16 | 0.96-1.40 | 1.16 | 0.96-1.41 |
| GCSE or equivalent |  |  | 1.28 | 1.08-1.51 | 1.11 | 0.93-1.32 | 1.10 | 0.92-1.31 | 1.14 | 0.95-1.36 |
| Foreign/other qualification |  |  | 1.02 | 0.68-1.51 | 0.98 | 0.65-1.50 | 1.01 | 0.67-1.55 | 1.10 | 0.71-1.70 |
| No qualification |  |  | 1.42 | 1.16—1.73 | 1.14 | 0.93-1.42 | 1.13 | 0.92-1.40 | 1.26 | 1.02-1.56 |
|  |  |  |  |  |  |  |  |  |  |  |
| Area-level deprivation (IMD quintiles) |  |  |  |  |  |  |  |  |  |  |
| 1 (least deprived) |  |  | 1 |  | 1 |  | 1 |  | 1 |  |
| 2 |  |  | 1.16 | 0.95-1.38 | 1.07 | 0.88-0.31 | 1.07 | 0.88-1.31 | 1.05 | 0.86-1.29 |
| 3 |  |  | 1.39 | 1.13-1.71 | 1.20 | 0.97-1.49 | 1.19 | 0.96-1.48 | 1.23 | 0.98-1.53 |
| 4 |  |  | 1.31 | 1.08-1.59 | 1.12 | 0.92-1.38 | 1.10 | 0.90-1.35 | 1.12 | 0.91-1.39 |
| 5 (most deprived) |  |  | 1.62 | 1.33-1.98 | 1.27 | 1.03-1.58 | 1.29 | 1.03-1.59 | 1.31 | 1.05-1.63 |
|  |  |  |  |  |  |  |  |  |  |  |
| Common mental disorder |  |  |  |  | 6.93 | 6.07-7.91 | 6.96 | 6.09-7.95 | 6.03 | 5.26-6.91 |
| Past-year discrimination on grounds of sexual orientation |  |  |  |  |  |  | 1.87 | 0.92-3.80 |  |  |
| Lifetime history of being bullied |  |  |  |  |  |  |  |  | 2.95 | 2.59-3.35 |

**Supplementary Table 5: Associations between sexual orientation and lifetime suicide attempt (*post hoc* analysis)**

|  | **Unadjusted association** | | **Adjusted for socio-demographic factors** | | **Adjusted for socio-demographic factors and CMD** | | **Model 3 adjusted for discrimination** | | **Model 3 adjusted for bullying** | |
| --- | --- | --- | --- | --- | --- | --- | --- | --- | --- | --- |
|  | **Model 1** | | **Model 2** | | **Model 3 (final model)** | | **Model 4** | | **Model 5** | |
|  | OR | 95% CI | OR | 95% CI | OR | 95% CI | OR | 95% CI | OR | 95% CI |
| **Lifetime suicide attempt** | N=10,433 |  | N=10,319 |  | N=10,319 |  | 10,250 |  | N=10,317 |  |
| **Sexuality** |  |  |  |  |  |  |  |  |  |  |
| Heterosexual | 1 |  | 1 |  | 1 |  | 1 |  | 1 |  |
| Bisexual | **5.54** | **3.44-8.91** | **4.75** | **2.88-7.83** | **3.55** | **2.05-6.16** | **3.15** | **1.77-5.61** | **3.23** | **1.89-5.50** |
| Lesbian / Gay | **3.12** | **1.91-5.08** | **3.7** | **2.20-6.22** | **3.26** | **1.87-5.69** | **2.22** | **1.10-4.52** | **2.64** | **1.50-4.66** |
| Other | 1.7 | 0.92-3.15 | 1.38 | 0.73-2.63 | 1.20 | 0.58-2.42 | 1.18 | 0.58-2.44 | 1.21 | 0.59-2.45 |
|  |  |  |  |  |  |  |  |  |  |  |
| Year 2007 |  |  | 1 |  | 1 |  | 1 |  | 1 |  |
| Year 2014 |  |  | 1.23 | 1.01-1.49 | 1.18 | 0.96-1.44 | 1.16 | 0.94-1.42 | 1.14 | 0.93-1.41 |
|  |  |  |  |  |  |  |  |  |  |  |
| Male gender |  |  | 0.63 | 0.53-0.77 | 0.92 | 0.63-0.94 | 0.76 | 0.62-0.92 | 0.75 | 0.63-0.95 |
| Age |  |  | 1 | 0.99-1 | 1 | 0.99-1 | 1 | 0.99-1.01 | 1 | 0.99-1.01 |
|  |  |  |  |  |  |  |  |  |  |  |
| Qualifications |  |  |  |  |  |  |  |  |  |  |
| Degree |  |  | 1 |  | 1 |  | 1 |  | 1 |  |
| Teaching, HND, Nursing |  |  | 1.78 | 1.18-2.70 | 1.69 | 01.10-2.59 | 1.67 | 1.09-2.58 | 0.70 | 1.11-2.61 |
| A level |  |  | 1.52 | 1.09-2.13 | 1.38 | 0.98-1.95 | 1.37 | 0.97-1.93 | 1.40 | 0.98-2 |
| GCSE or equivalent |  |  | 2.24 | 1.67-3 | 1.97 | 1.46-2.67 | 1.95 | 1.4-2.64 | 2.05 | 1.50-2.80 |
| Foreign/other qualification |  |  | 1.27 | 0.66-2.4 | 1.24 | 0.65-2.39 | 1.27 | 0.67-2.45 | 1.36 | 0.68-2.73 |
| No qualification |  |  | 2.8 | 2-3.93 | 2.31 | 1.63-3.29 | 2.21 | 1.58-3.08 | 2.57 | 1.79-3.67 |
|  |  |  |  |  |  |  |  |  |  |  |
| Area-level deprivation (IMD quintiles) |  |  |  |  |  |  |  |  |  |  |
| 1 (least deprived) |  |  | 1 |  | 1 |  | 1 |  | 1 |  |
| 2 |  |  | 0.87 | 0.61-1.24 | 0.80 | 0.55-1.15 | 0.81 | 0.56-1.17 | 0.77 | 0.53-1.11 |
| 3 |  |  | 1.55 | 1.09-2.21 | 1.31 | 0.91-1.89 | 1.34 | 0.93-1.92 | 1.34 | 0.93-1.93 |
| 4 |  |  | 1.40 | 0.99-1.97 | 1.17 | 0.82-1.67 | 1.15 | 0.81-1.64 | 1.18 | 0.83-1.67 |
| 5 (most deprived) |  |  | 2.10 | 1.50-2.95 | 1.65 | 1.15-2.35 | 1.70 | 1.20-2.41 | 1.68 | 1.18-2.40 |
|  |  |  |  |  |  |  |  |  |  |  |
| Common mental disorder |  |  |  |  | 6.00 | 4.94-7.29 | 6.04 | 4.97-7.35 | 5.04 | 4.11-6.16 |
| Past-year discrimination on grounds of sexual orientation |  |  |  |  |  |  | 3.38 | 1.45-7.89 |  |  |
| Lifetime history of being bullied |  |  |  |  |  |  |  |  | 2.61 | 2.11-3.21 |

**Supplementary Table 6: Models with interactions between sexual orientation and gender**

|  | **Past-year suicidal thoughts (N=10,433)** | | **Past-year suicide attempts (N=10,433)** | | **Lifetime NSSH (N=10,432)** | |
| --- | --- | --- | --- | --- | --- | --- |
|  | **OR** | **95% CI** | **OR** | **95% CI** | **OR** | **95% CI** |
| Heterosexual | 1 |  | 1 |  | 1 |  |
| Bisexual | 3.00 | 1.52-5.92 | 6.54 | 1.94-22.06 | 7.04 | 4.16-11.91 |
| Lesbian / Gay | 2.35 | 0.84-6.57 | 2.89 | 0.85-9.88 | 5.43 | 2.83-10.40 |
| Other | 1.17 | 0.50-2.77 | 0.56 | 0.08-4.14 | 1.49 | 0.68-3.23 |
|  |  |  |  |  |  |  |
| Male gender | 0.76 | 0.62-0.94 | 0.70 | 0.39-1.26 | 0.75 | 0.61-0.93 |
|  |  |  |  |  |  |  |
| Bisexual*male | 0.71 | 0.17-3.07 | 0.46 | 0.04-4.77 | 0.58 | 0.18-1.87 |
| Lesbian / Gay*male | 1.17 | 0.33-4.15 | 0.28 | 0.03-2.95 | 0.45 | 0.17-1.17 |
| Other*male | 2.49 | 0.77-8.10 | 8.57 | 0.64-115.23 | 1.60 | 0.41-6.33 |
|  | **p-value** |  | **p-value** |  | **p-value** |  |
| Overall p-value for interaction | 0.462 |  | 0.214 |  | 0.251 |  |

**Supplementary Table 7: Models with interactions between sexual orientation and year**

|  | **Past-year suicidal thoughts (N=10,433)** | | **Past-year suicide attempts (N=10,433)** | | **Lifetime NSSH (N=10,432)** | |
| --- | --- | --- | --- | --- | --- | --- |
|  | **OR** | **95% CI** | **OR** | **95% CI** | **OR** | **95% CI** |
| Heterosexual | 1 |  | 1 |  | 1 |  |
| Bisexual | 3.22 | 1.17-8.90 | 13.95 | 3.79-51.42 | 3.49 | 1.19-10.18 |
| Lesbian / Gay | 1.69 | 0.71-4.06 | 2.03 | 0.46-9.02 | 3.81 | 1.93-7.53 |
| Other | 2.17 | 1.01-4.69 | 3.01 | 0.61-14.79 | 1.29 | 0.46-3.59 |
|  |  |  |  |  |  |  |
| Year 2007 | 1 |  | 1 |  | 1 |  |
| Year 2014 | 1.18 | 0.96-1.45 | 1.08 | 0.62-1.88 | 1.68 | 1.38-2.06 |
|  |  |  |  |  |  |  |
| Bisexual*2014 | 0.84 | 0.24-3.01 | 0.17 | 0.02-1.85 | 2.18 | 0.65-7.30 |
| Lesbian / Gay*2014 | 1.72 | 0.54-5.45 | 0.55 | 0.07-4.47 | 0.71 | 0.28-1.80 |
| Other*2014 | 0.69 | 0.21-2.32 | 0.30 | 0.02-3.92 | 2.08 | 0.54-8.02 |
|  | **p-value** |  | **p-value** |  | **p-value** |  |
| Overall p-value for interaction | 0.727 |  | 0.382 |  | 0.330 |  |
